# Comparison of antiplatelet regimens in secondary stroke prevention: a nationwide cohort study

**DOI:** 10.1186/s12883-015-0480-4

**Published:** 2015-11-02

**Authors:** Christine Benn Christiansen, Jannik Pallisgaard, Thomas Alexander Gerds, Jonas Bjerring Olesen, Mads Emil Jørgensen, Anna Karin Numé, Nicholas Carlson, Søren Lund Kristensen, Gunnar Gislason, Christian Torp-Pedersen

**Affiliations:** Aalborg University Hospital, Forskningens Hus, Sdr. Skovvej 15, 9000 Aalborg, Denmark; Department of Cardiology, Gentofte Hospital, Gentofte, Denmark; Department of Public Health, Section of Biostatistics, University of Copenhagen, Copenhagen, Denmark; National Institute of Public Health, University of Southern Denmark, Copenhagen, Denmark; Faculty of Health and Medical Sciences, University of Copenhagen, Copenhagen, Denmark; Department of Health Science and Technology, Aalborg University, Aalborg, Denmark

**Keywords:** Ischemic stroke, Secondary prevention, Antiplatelet, Epidemiology

## Abstract

**Background:**

In patients with ischemic stroke of non-cardioembolic origin, acetylsalicylic acid, clopidogrel, or a combination of acetylsalicylic acid and dipyridamole are recommended for the prevention of a recurrent stroke. The purpose of this study was to examine the risk of bleeding or recurrent stroke associated with these three treatments.

**Methods:**

Patients who were discharged with first-time ischemic stroke from 2007–2010, with no history of atrial fibrillation were identified from Danish nationwide registries. Hazard ratios (HRs) and 1-year risks of recurrent ischemic stroke and bleeding were calculated for each antiplatelet regimen.

**Results:**

Among patients discharged after first-time ischemic stroke, 3043 patients were treated with acetylsalicylic acid, 12,295 with a combination of acetylsalicylic acid and dipyridamole, and 3885 with clopidogrel. Adjusted HRs for clopidogrel versus the combination of acetylsalicylic acid and dipyridamole were 1.02 (95 % confidence interval [CI]: 0.89–1.17) for ischemic stroke and 1.06 (95 % CI: 0.83–1.35) for bleeding. Adjusted HRs for acetylsalicylic acid versus the combination of acetylsalicylic acid and dipyridamole were 1.48 (95 % CI: 1.31–1.67) for stroke and 1.47 (95 % CI: 1.18–1.82) for bleeding. Clopidogrel versus acetylsalicylic acid yielded HRs of 0.69 (95 % CI: 0.59–0.81) and 0.72 (95 % CI: 0.55–0.96) for stroke and bleeding, respectively. The 1-year predicted risks associated with acetylsalicylic acid, the combination of acetylsalicylic acid and dipyridamole, and clopidogrel were 11.1 (95 % CI: 10.2–12.2), 7.7 (95 % CI: 7.3–8.3), and 8.0 (95 % CI: 6.9–8.7) for ischemic stroke, respectively; while, the risks for bleeding were 3.4 (95 % CI: 2.8–3.9), 2.4 (95 % CI: 2.1–2.7), and 2.4 (95 % CI: 1.9–2.9), respectively.

**Conclusion:**

Clopidogrel and the combination of acetylsalicylic acid and dipyridamole were associated with similar risks for recurrent ischemic stroke and bleeding; whereas acetylsalicylic acid was associated with higher risks for both ischemic stroke and bleeding. The latter finding may partially be explained by selection bias.

## Background

After ischemic stroke or transient ischemia attack, patients are at high risk for a subsequent stroke. In the Unites States, 25 % of the 795,000 annual ischemic strokes are recurrent events [1] and 70–84 % of ischemic stroke are non-cardioembolic [2, 3]. International guidelines recommend antiplatelets for secondary prevention in patients with strokes of non-cardioembolic origin [1, 4]. The recommended treatment regimens include acetylsalicylic acid, a combination of acetylsalicylic acid and dipyridamole, or clopidogrel.

Large-scale clinical randomized studies have evaluated the efficacy and safety of the recommended treatments. The combination of acetylsalicylic acid and dipyridamole was reported to be more effective than acetylsalicylic acid alone in preventing subsequent stroke, and may be associated with a decreased risk of bleeding [5, 6]. In patients with ischemic stroke, no significant difference was found between clopidogrel and acetylsalicylic acid for the prevention of ischemic stroke/myocardial infarction (MI)/cardiovascular (CV) death [7]. Treatment with clopidogrel was as effective in preventing ischemic stroke as the combination of acetylsalicylic acid and dipyridamole, and was associated with a lower risk of bleeding [8]. Since the combination of acetylsalicylic acid and dipyridamole was associated with a lower risk of recurrent stroke than acetylsalicylic acid alone and clopidogrel was not significantly different from acetylsalicylic acid, it was surprising that there was no significant difference between clopidogrel and the combination of acetylsalicylic acid and dipyridamole. This paradox may be due to differences between study designs, such as inclusion criteria and drug dosage [9].

Epidemiological research in a real-world cohort can provide additional evidence of the efficacy and safety of antiplatelet regimens, and thereby help to guide future guidelines on therapy. Therefore, using data from Danish nationwide registries, we investigated the risk of recurrent stroke and bleeding in patients with ischemic stroke from January 1^st^ 2007 to December 31^st^ 2010. We compared patients treated with acetylsalicylic acid, the combination of acetylsalicylic acid and dipyridamole, and clopidogrel.

## Methods

### Registries

Data were accessed and linked through encrypted identification numbers using registries in Statistics Denmark. All Danish citizens carry a unique civil registration number and the registries cover the entire Danish population. Diagnoses were found in the National Patient Register, where diagnoses from hospital discharges are registered according to the International Classification of Diseases (ICD) with ICD-8 codes from 1978 and ICD-10 codes from 1995 [10]. Information on medication was found in the Danish Medicinal Register, where filled prescriptions were registered since 1994 according to the Anatomical Therapeutic Chemical (ATC) Classification System [11]. High degree of validity for these data has been reported previously [12]. The cause and date of death was retrieved from the Danish Register of Causes of Death [13].

### Study population and antiplatelet therapy

All patients discharged with first-time ischemic stroke (ICD-8: 433, 434, 436 or ICD-10: I63, I64) in the study period from January 1^st^ 2007-December 31^st^ 2010 were eligible for inclusion. Exclusion depended on the presence of the following before or up to 30 days after discharge: atrial fibrillation (ICD-8 code: 42793–42794, ICD-10: I48) at baseline or anticoagulation therapy (ATC: B01AA, B01AE07, B01AF02, B01AF01). Acetylsalicylic acid was defined by ATC-code B01AC06 or N02BA01 and clopidogrel was defined by ATC-code B01AC04. The combination of acetylsalicylic acid and dipyridamole was defined by either ATC-code B01AC30 or redeemed prescriptions for both acetylsalicylic acid and dipyridamole (ATC-code B01AC07). Patients that did not receive either acetylsalicylic acid, a combination of dipyridamole and acetylsalicylic acid, or clopidogrel at baseline were excluded. Acetylsalicylic acid may be sold as an over the counter drug in Denmark, in which case the purchase is not registered. However, prescription of the drug is required to obtain reimbursement, which enables linkage to an individual. Prescription is required for the acquisition of dipyridamole, clopidogrel, and glucose-lowering drugs.

### Comorbidities and concomitant medication

Diabetes mellitus, heart failure, and renal disease were defined as previously [14, 15]: diabetes mellitus by the use of glucose-lowering drugs (ATC: A10) and heart failure (ICD-8: 425, 4270, 4271; ICD-10: I110, I42, I50, J819) and renal disease by diagnosis (ICD-8: 24902, 25002, 40399, 58000, 58199, 582–3, 59009, 591, 75234; ICD-10: E102, E112, E132, E142, I120, N02-8, N11-12, N14, N18-9, N26, N158-160, N162-4, N168, Q612-3, Q615, Q619). The discharge diagnosis prior to study inclusion was also used to define MI (ICD-8: 410; ICD-10: I21, I22), peripheral artery disease (ICD-8: 440; ICD-10 DI700, I702-9), chronic obstructive pulmonary disease (ICD-8: 491–2; ICD-10: J42-4), and cancer (ICD-8 140–218 or ICD-10 codes C). Bleeding was defined as gastrointestinal bleeding (ICD-8: 53190, 53192, 53195, 53290, 53390, 53490, 53501, 56915; ICD-10: K250, K252 K254, K256, K260, K262, K264, K266, K270, K272, K274, K276, K920-2), intracranial hemorrhage, or bleeding from the urogenital or respiratory system (ICD-8: 28001,43008-9,43098-9, 431; ICD-10 I60-62, N02, R04, R31, I690-2, J942) as done previously [15]. Hypertension was defined by concomitant use of medication from at least two of the following classes of antihypertensive drugs: antiadrenergics, diuretics, vasodilators, beta-blockers, calcium channel blockers, and renin angiotensin converting enzyme inhibitors at baseline. This definition of hypertension has been validated and has a specificity of 94.7 % [14, 16].

### Outcomes

The following outcomes of interest were investigated: diagnoses of fatal or nonfatal ischemic stroke and fatal or nonfatal bleeding.

### Study design

All patients with first-time ischemic stroke between January 1^st^ 2007 and December 31^st^ 2010 were identified. Data on outcomes until December 31^st^ 2011 were available. Patients were included if they survived the first 30 days after stroke. Follow-up commenced 30 days after discharge to allow time to redeem prescriptions, and ended when patients experienced an outcome of interest, died, emigrated, or one year after discharge, whichever came first.

### Statistical analysis

*P*-values for baseline characteristics were calculated with Chi-square test for categorical variables and Wilcoxon for continuous variables. HR of ischemic stroke and bleeding were calculated based on cause-specific Cox regression analyses. These analyses were adjusted for sex, age, year, prior myocardial infarction, hypertension, diabetes, peripheral artery disease, heart failure, chronic obstructive lung disease and cancer. The proportional hazard assumption was evaluated by score process tests [17].

The absolute risks of stroke (bleeding) within 1 year were calculated by combining the cause-specific Cox regression analysis for stroke (bleeding) with a second cause-specific Cox regression analysis for the competing risks. The latter was adjusted for the confounders listed above and also included the factual treatment. We predicted a personalized absolute 1-year risk of stroke (bleeding) for each patient in counter factual data were the treatment variable was set to acetylsalicylic acid (clopidogrel, acetylsalicylic acid/dipyridamole) for all patients but keeping the factual values of all confounders. Reported were mean 1-year risks of stroke (bleeding) across all patients associated with acetylsalicylic acid (clopidogrel, acetylsalicylic acid/dipyridamole) with bootstrap confidence limits (CI) based on 200 bootstrap samples drawn with replacement from the full data. All programming and analyses were run in SAS 9.4 (SAS Institute Inc., Gary, NC, USA) and R 3.0.2. (R Core Team (2013). R: A language and environment for statistical computing. (R Foundation for Statistical Computing, Vienna, Austria).

### Ethics

Ethical approval is not required for historical register-based studies in Denmark The study is consistent with the Declaration of Helsinki and was approved by the Danish Data Protection Agency (ref. 2007-58-0015, local journal number GEH 2014–013, I-suite number 02731). As the civil registration numbers of the patients were encrypted, individuals could not be identified. The authors take full responsibility for the integrity of data.

## Results

### Study population

**Fig. 1:**
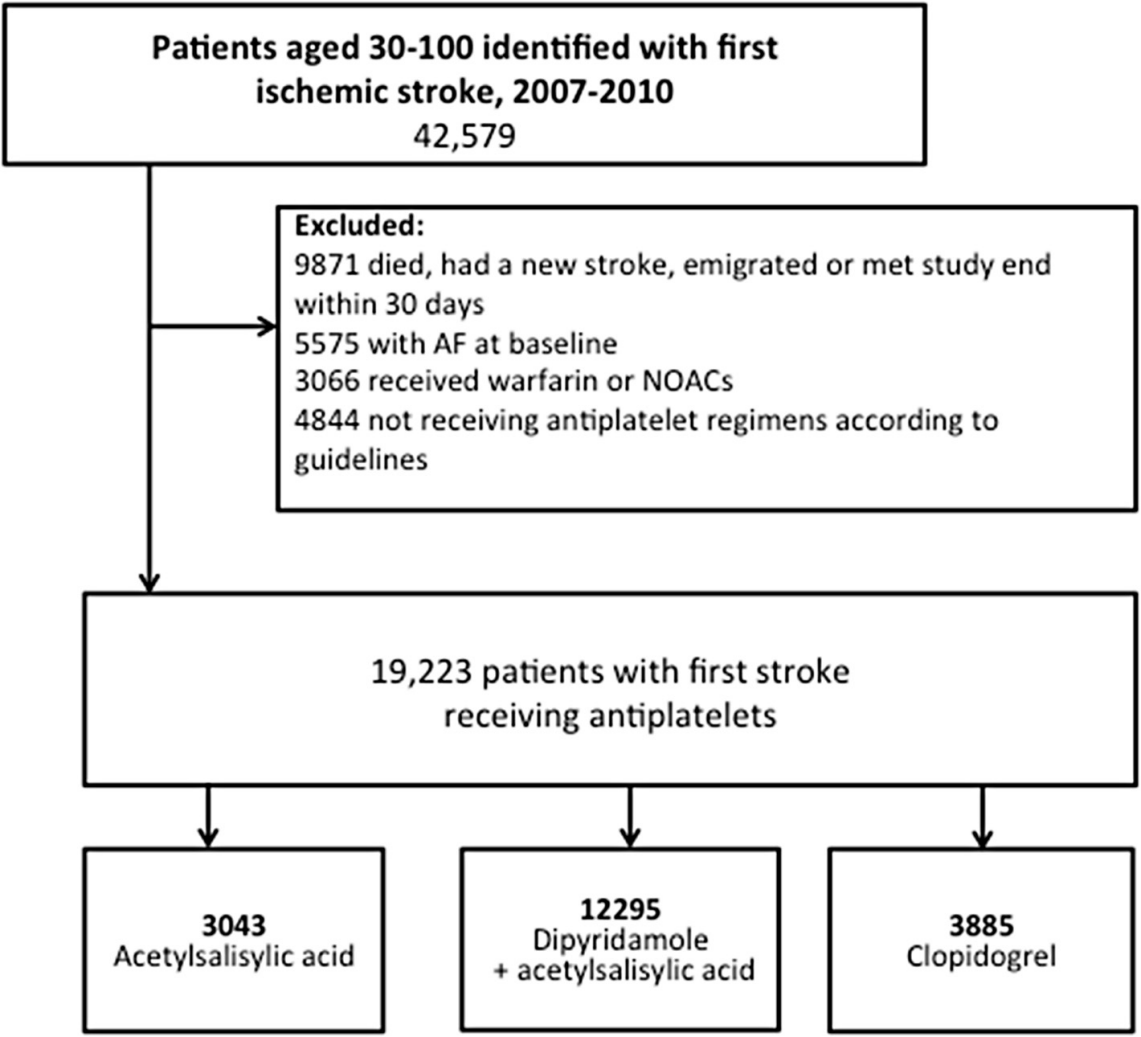
Flowchart of study population

A flowchart of the study population is shown in Fig. 1. Of the 42,295 patients included after a first-time ischemic stroke, 19,223 remained after exclusion of patients with atrial fibrillation, those taking oral anticoagulants, and patients not receiving any antithrombotic treatment. Of the included patients, 3043 were treated with acetylsalicylic acid, 12,295 with acetylsalicylic acid and dipyridamole, and 3885 with clopidogrel. Baseline characteristics are summarized in Table 1. Patients with acetylsalicylic acid alone were older (median age 75.3 years, interquartile range [IQR, 64.4–83.7]) and less often male (47.5 %) than patients treated with the combination of acetylsalicylic acid and dipyridamole (median age 70.7 years [IQR, 61.5–79.6], 56.5 % males) or clopidogrel (median age 68.6 years [IQR, 59.2–77.6], 49.3 % males). Patients receiving acetylsalicylic acid alone had more hypertension, renal disease, chronic obstructive pulmonary disease, and previous bleeding than patients treated with the combination of acetylsalicylic acid and dipyridamole or clopidogrel. Patients treated with the combination of acetylsalicylic acid and dipyridamole had less MIs then patients receiving acetylsalicylic acid and clopidogrel. The median follow-up time was 335 days [IQR, 335–335] for all treatment groups and the total follow-up time was 2475, 10,601, and 3364 person-years for acetylsalicylic acid, combination of acetylsalicylic acid and dipyridamole, and clopidogrel, respectively. The corresponding number of ischemic strokes was 360, 957 and 291 and the number of bleedings was 123, 286 and 92. Nine people emigrated; one person receiving clopidogrel, to receiving acetylsalicylic acid and six on combination therapy.

**Table 1:** Baseline characteristics

	Acetylsalicylic acid	Acetylsalicylic acid + dipyridamole	Clopidogrel	*p*-value
	(*n* = 3043)	(*n* = 12,295)	(*n* = 3885)	
Age in years, median (IQR)	75.3 (64.4–83.7)	70.7 (61.5–79.6)	68.6 (59.2–77.6)	>0.0001
Men (%)	47.5 %	56.5 %	49.3 %	>0.0001
Diabetes	380 (12.5 %)	1449 (11.8 %)	455 (11.7 %)	0.5263
Hypertension	1316 (43.2 %)	4987 (40.6 %)	1644 (42.3 %)	0.0102
Myocardial infarction	334 (11 %)	824 (6.7 %)	523 (13.5 %)	<0.01
Peripheral artery disease	126 (4.1 %)	297 (2.4 %)	180 (4.6 %)	<0.01
Heart failure	222 (7.3 %)	496 (4 %)	229 (5.9 %)	<0.01
Cancer	206 (6.8 %)	714 (5.8 %)	245 (6.3 %)	0.1062
Chronic obstructive pulmonary disease	256 (8.4 %)	808 (6.6 %)	274 (7.1 %)	<0.01
Previous bleeding	405 (13.3 %)	853 (6.9 %)	319 (8.2 %)	<0.01
NSAIDS	485 (15.9 %)	1751 (14.2 %)	600 (15.4 %)	0.0243

### Time trends in antiplatelet regimens after ischemic stroke

**Fig. 2:**
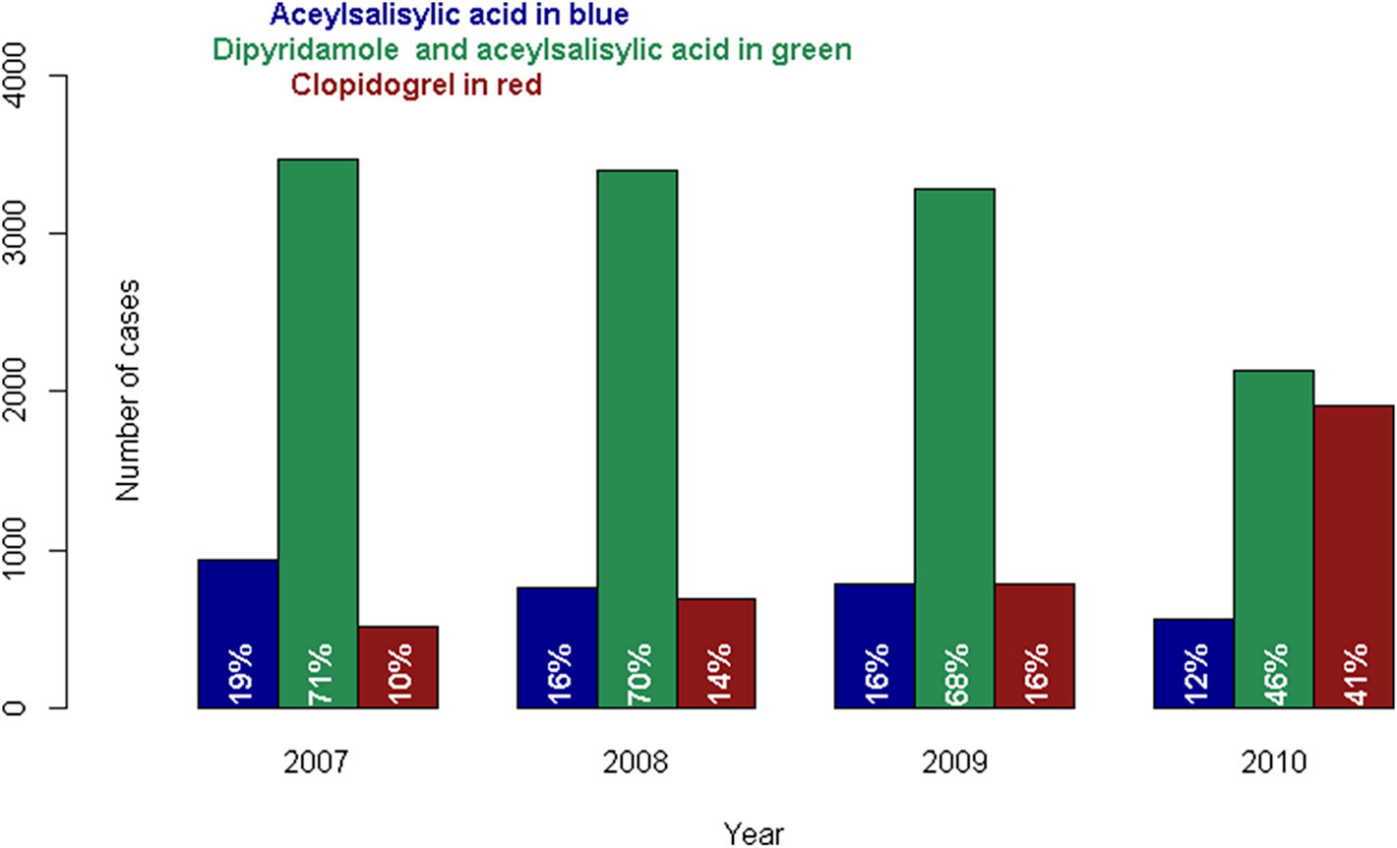
Trends in treatments after first-time ischemic stroke from 2007 through 2010: Number of cases on each antiplatelet regimen

The proportion of patients with ischemic stroke assigned to the different antiplatelet regimens during the study period is shown in Fig. 2. From 2007 to 2010, the fraction of patients that received acetylsalicylic acid and the combination of acetylsalicylic acid and dipyridamole decreased from 19 to 12 % and from 71 to 46 %, respectively; whereas, the proportion of patients that received clopidogrel increased from 10 to 41 %.

### Adherence to treatment

The adherence to the antiplatelet regimen at baseline is shown in Table 2. The proportions of patients (alive and event free) that adhered to the baseline antiplatelet regimens were 86.2, 91.3, and 92.8 %/89.2 % for acetylsalicylic acid, clopidogrel, and acetylsalicylic acid/dipyridamole, respectively.

**Table 2:** Adherence to baseline drugs. Proportion of patients that redeemed prescriptions for each antiplatelet treatment at 6 and 12 months after first ischemic stroke

Baseline treatment	Proportion of patients that redeemed a prescription after 6 months of baseline drug (%)	Proportion of patients that redeemed a prescription after 12 months (%)
Clopidogrel	91.3	87.5
Acetylsalicylic acid and dipyridamole	92.8 (acetylsalicylic acid)	91.7 (acetylsalicylic acid)
	89.2 (dipyridamole)	87.3 (dipyridamole)
Acetylsalicylic acid	86.0	85.6

### Risk of stroke and bleeding

**Fig. 3:**
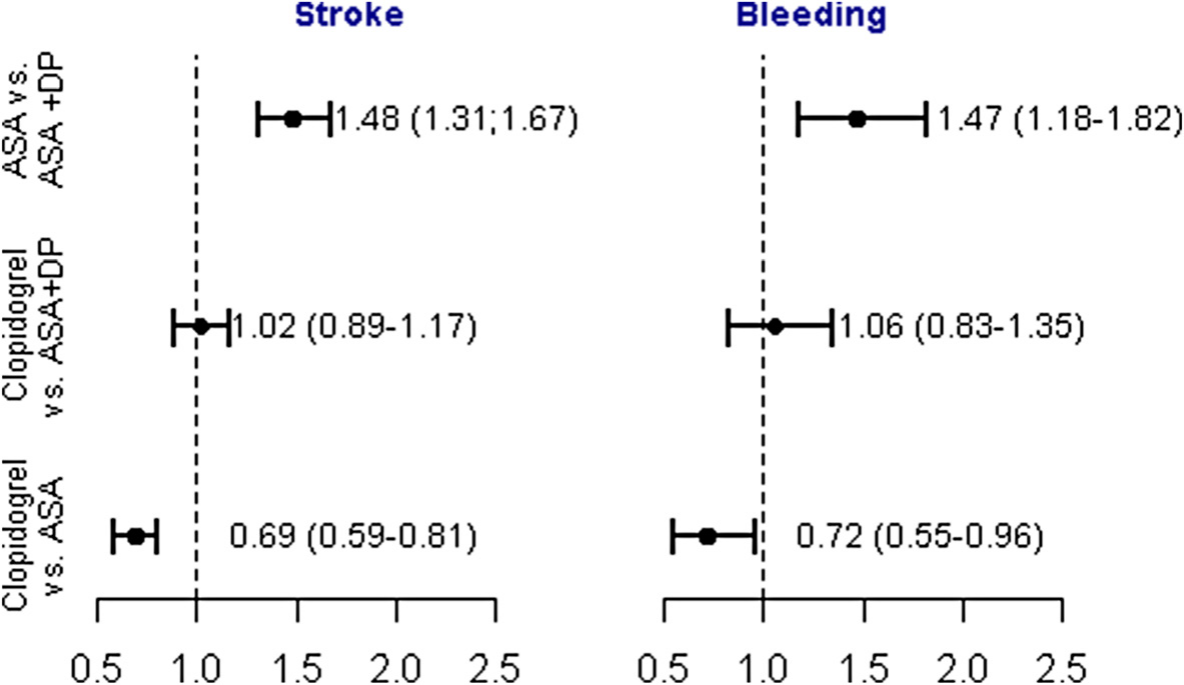
Results from cause- specific Cox regression analysis: cause-specific hazard ratios for ischemic stroke and bleeding. Other death denotes any death other than cardiovascular death. Abbreviations: ASA = acetylsalicylic acid and DP = dipyridamole. All endpoints are adjusted for sex, age, prior myocardial infarction, hypertension, diabetes, peripheral artery disease, heart failure, chronic obstructive lung disease, and cancer. Bleeding is further adjusted for previous bleeding

Adjusted HRs of ischemic stroke and bleeding are shown in Fig. 3. No significant difference was found for clopidogrel versus the combination of acetylsalicylic acid and dipyridamole for stroke (HR 1.02 [95 % CI: 0.89–1.17]) or bleeding (HR 1.06 [95 % CI: 0.83–1.35]). Acetylsalicylic acid was associated with significantly increased HRs for both endpoints when compared with the combination of acetylsalicylic acid and dipyridamole, and clopidogrel was associated with decreased HRs for ischemic stroke and bleeding when compared with acetylsalicylic acid.

Absolute risks of ischemic stroke and bleeding are shown in Table 3. These results take into account the competing risk of death for reasons other than ischemic stroke and bleeding, and are associated with settings where all patients would be treated with acetylsalicylic acid (clopidogrel, acetylsalicylic acid/dipyridamole). At 3, 6, and 12 months, the risks of ischemic stroke and bleeding were similar for clopidogrel and combination of acetylsalicylic acid and dipyridamole. The acetylsalicylic acid group had generally high risks of bleeding and ischemic stroke.

**Table 3:** Predicted 1-year risk of ischemic stroke and bleeding based on Cox regression analysis

	Risk of ischemic stroke			Risk of bleeding		
	3 months after stroke	6 months after stroke	12 months after stroke	3 months after stroke	6 months after stroke	12 months after stroke
Acetylsalicylic acid	3.6 [3.2; 4.0]	6.9 [6.3;7.7]	11.1 [10.2; 12.2]	1.2 [0.9; 1.4]	2.0 [1.6; 2.4]	3.4 [2.8; 3.9]
Acetylsalicylic acid and dipyridamole	2.4 [2.2; 2.7]	4.7 [4.4; 5.2]	7.7 [7.3; 8.3]	0.8 [0.7; 0.9]	1.4 [1.2; 1.6]	2.4 [2.1; 2.7]
Clopidogrel	2.5 [2.2; 2.9]	4.9 [4.3; 5.4]	8.0 [6.9; 8.7]	0.8 [0.6; 1.0]	1.4 [1.0; 1.7]	2.4 [1.9; 2.9]

The model was adjusted for sex, age, year, prior myocardial infarction, hypertension, diabetes, peripheral artery disease, heart failure, chronic obstructive lung disease, and cancer

## Discussion

This historical register-based nationwide cohort study comprised more than 19,000 cases of first-time ischemic stroke without atrial fibrillation and treated with antiplatelet regimens. The risks of ischemic stroke and bleeding were similar for the combination of acetylsalicylic acid and dipyridamole and for clopidogrel; whereas, acetylsalicylic acid alone was associated with higher risks of both ischemic stroke and bleeding.

### Acetylsalicylic acid versus the combination of acetylsalicylic acid and dipyridamole

The European Stroke Prevention Study 2 (ESPS 2, *n* = 3299 with ischemic stroke or TIA within 3 months) found that the combination of acetylsalicylic acid and dipyridamole was more efficient than acetylsalicylic acid alone in preventing strokes. On the other hand, the European/Australasian Stroke Prevention in Reversible Ischemia Trial (ESPRIT) (*n* = 2739 patients with stroke or TIA within 6 months) did not show significant reduction in the risk of ischemic stroke or all-cause death, but did find significantly decreased risk for the combined endpoint of ischemic stroke/MI/CV death with the combination therapy [5]. Of 1376 patients treated with the combination of acetylsalicylic acid and dipyridamole, 470 patients discontinued treatment, compared with 184 in the acetylsalicylic acid group.

Bleeding was not an endpoint in ESPS 2, but similar proportions of bleeding were reported as adverse events in the acetylsalicylic acid and combination of acetylsalicylic acid and dipyridamole groups (135/1649 and 144/1650, respectively. ESPRIT reported a decreased risk of major bleeding (defined as intracranial bleeding, any fatal bleeding, or bleedings requiring hospital admission) for the combination of acetylsalicylic acid and dipyridamole in the on treatment group. The investigators explained this finding as a play of chance.

### Selection bias

In an observational study, there is a risk of confounding by indication and selection bias since the study population is not randomized at baseline. In our study the risk of other death (other than fatal stroke) was increased in the acetylsalicylic acid group compared with the combination group, which indicates a difference between the two groups. As CV death overall and not just ischemic stroke may be influenced by antiplatelet treatment, we performed a sensitivity analysis of the combined endpoint of stroke/MI/CV death (data not shown), where “other death” did not include MI or CV death and hence should not be influenced by antiplatelet treatment. However, also here the HR of “other death” remained increased for acetylsalicylic acid even after adjusting for comorbidity. This indicates that treatment with acetylsalicylic acid was associated with factors that led to poorer outcome and that this difference was not explained by the available data on comorbidity. Hence, all results regarding acetylsalicylic acid are likely negatively biased and should be interpreted with great care.

### Clopidogrel versus acetylsalicylic acid

The Clopidogrel versus Aspirin in Patients at Risk of Ischemic Events (CAPRIE) study included patients with previous stroke, MI, or peripheral artery disease [7]. A significantly decreased risk of stroke/MI/CV death was reported for clopidogrel versus acetylsalicylic acid in the study population overall, but no decrease in risk was found for the sub-group analysis of patients included after ischemic stroke. In contrast to our findings, the higher risk of overall bleeding was not increased in CAPRIE. This discrepancy may be explained by selection bias, similar to what was described above for the combination of acetylsalicylic acid and dipyridamole versus acetylsalicylic acid.

### Clopidogrel versus acetylsalicylic acid and dipyridamole

The finding that clopidogrel is associated with a similar risk as the combination of acetylsalicylic acid and dipyridamole for ischemic stroke and the combined endpoint of stroke/MI/CV death is in accordance with results from the Prevention Regimen for Effectively avoiding Second Strokes (PRoFESS), where patients could be included up to 120 days after the qualifying stroke (*n* = 20,332) [8]. PRoFESS reported no significant difference in the risk of hemorrhagic events, but did report increased risk of *major* bleeding (bleeding causing hospitalizations) in patients treated with the combination of acetylsalicylic acid and dipyridamole compared with clopidogrel recipients (HR 1.15 [95 % CI: 1.00–1.32]). Differences in definitions of bleeding or statistical chance may also explain the difference in results. The network meta-analysis of ESPS2, ESPRIT, CAPRIE, and PRoFESS addressed the apparant paradox of clopidogrel being equal to the combination of acetylsalicylic acid and dipyridamole as well as to acetylsalicylic acid, when the combination of acetylsalicylic acid and dipyridamole was superior to acetylsalicylic acid alone. The analysis concluded that the risk of recurrence stroke was not significantly different between the three antiplatelet regimens [9].

### Strengths and limitations

The study was a register-based observational study with built-in limitations that should be acknowledged. Due to the risk of selection bias, the most important limitation is that we do not have any data explaining the choice of antiplatelets, such as intolerance. Another important limitation is that we did not have access to data on stroke or bleeding severity. Minor bleedings remained unidentified in case they did not cause admissions to hospital. Minor bleedings remained unidentified in case they did not cause admissions to hospital. If the unregistered bleedings were equally distributed between treatment groups, the actual relative difference would be smaller, while the absolute difference would not be affected. In addition, we did not have data on the smoking status, body mass index, exercise, dosage of acetylsalicylic acid, or the diagnoses registered by general practitioners, and this may influence the results. The strengths include the size of the study with the advantage that data on hospitalizations and medication for the entire Danish population were available. The registration of the diagnosis of stroke has a high sensitivity and specificity [18].

### Generalizability

This study is representative for the Danish population and results may plausibly apply to similar Western European countries. In other populations with different ages, ethnicities, socioeconomic and lifestyle factors, the stroke etiology may differ, which may affect the impact of antiplatelet therapy.

### Implications and further studies

Our results indicate that clopidogrel or the combination of acetylsalicylic acid and dipyridamole are equal alternatives for the prevention of recurrent stroke in patients with ischemic stroke. As noted, due to the risk of selection bias, the results regarding acetylsalicylic acid should be interpreted with care.

## Conclusion

In this nationwide study comprising more than 19,000 patients with ischemic stroke, the proportion of patients with first time ischemic stroke that received clopidogrel increased during the study period from 2007–2010. The risk of secondary ischemic stroke as well as the risk of bleeding were similar for treatment with clopidogrel and the combination of acetylsalicylic acid and dipyridamole. Treatment with acetylsalicylic acid alone was associated with worse outcomes, but was not comparable to the other groups due to risk of bias.
